# Cyclophilin A as a Pro-Inflammatory Factor Exhibits Embryotoxic and Teratogenic Effects during Fetal Organogenesis

**DOI:** 10.3390/ijms241411279

**Published:** 2023-07-10

**Authors:** Anastasiia Kalinina, Maria Semenova, Alexandra Bruter, Ekaterina Varlamova, Marina Kubekina, Natalia Pavlenko, Yulia Silaeva, Alexey Deikin, Elena Antoshina, Tatyana Gorkova, Lubov Trukhanova, Alla Salmina, Svetlana Novikova, Dmitry Voronkov, Dmitry Kazansky, Ludmila Khromykh

**Affiliations:** 1 N.N. Blokhin National Medical Research Center of Oncology, Ministry of Health of the Russian Federation, 24 Kashirskoe Shosse, Moscow 115478, Russiatgorkova70@gmail.com (T.G.); trukhanova80@gmail.com (L.T.);; 2 Department of Embryology, Faculty of Biology, Moscow State University, 1/12 Leninskie Gory, Moscow 119992, Russia; 3 Center for Precision Genome Editing and Genetic Technologies for Biomedicine, Institute of Gene Biology, Russian Academy of Sciences, 34/5 Vavilov Street, Moscow 119334, Russia; 4 Core Facility Center, Institute of Gene Biology, Russian Academy of Sciences, 34/5 Vavilova Street, Moscow 119334, Russia; yulya.silaeva@gmail.com; 5 United Center for Genetic Technologies, Belgorod State National Research University, 85 Pobedi Street, Belgorod 308001, Russia; 6 Research Center of Neurology, 80 Volokolamskoye Shosse, Moscow 125367, Russia; allasalmina@mail.ru (A.S.); levik_82@mail.ru (S.N.);

**Keywords:** Cyclophilin A, pro-inflammatory factor, complicated pregnancy, miscarriage, embryotoxicity, teratogenicity, organogenesis, fetal anatomical defects, transgenic mice

## Abstract

The precise balance of Th1, Th2, and Th17 cytokines is a key factor in successful pregnancy and normal embryonic development. However, to date, not all humoral factors that regulate and influence physiological pregnancy have been completely studied. Our data here pointed out cyclophilin A (CypA) as the adverse pro-inflammatory factor negatively affecting fetal development and associated with pregnancy complications. In different mouse models in vivo, we demonstrated dramatic embryotoxicity and teratogenicity of increased CypA levels during pregnancy. Using generated transgenic models, we showed that CypA overexpression in fetal tissues induced the death of all transgenic fetuses and complete miscarriage. Administration of recombinant human CypA in a high dose to pregnant females during fetal organogenesis (6.5–11.5 dpc) exhibited teratogenic effects, causing severe defects in the brain and bone development that could lead to malformations and postnatal behavioral and cognitive disorders in the offspring. Embryotoxic and teratogenic effects could be mediated by CypA-induced up-regulation of M1 macrophage polarization via activation of the STAT1/3 signaling pathways. Here, we propose secreted CypA as a novel marker of complicated pregnancy and a therapeutic target for the correction of pregnancy complications.

## 1. Introduction

Reproductive immunology focuses on immunological processes that support healthy pregnancy and labor, as well as the discovery of pathogenic factors that interfere with physiological pregnancy. To date, the systemic and local (in the uterus, placenta, and fetal membranes) network of maternal immune cells and the broad spectrum of cytokines of T helpers 1, 2, and 17 (Th1, Th2, and Th17) [1,2] are recognized as the pivotal regulators of reproductive immunity, promoting embryo viability and development.

Cytokines regulate the maternal immunological balance to induce local inflammation in the first trimester, promoting implantation and placentation [3]; support immunological tolerance and maternal–fetal symbiosis during fetal development; and re-establish inflammatory conditions at the end of gestation to promote contractions, labor, and rejection of the placenta [4]. Dysregulations in the cytokine network are associated with human pregnancy complications, including miscarriage, preterm labor, preeclampsia, and fetal growth restriction [1,2,5]. Both the array and concentration of cytokines are critical for the normal embryo’s development. Elevated concentrations of pro-inflammatory cytokines (TNFα, IFNγ, IL-1β, IL-6, etc.) at the maternal–fetal interface could exhibit pronounced embryotoxicity and suppress the embryonic development, inducing its death [6,7,8,9,10].

To date, cytokines affecting pregnancy and the impact of embryotoxic cytokines on embryo development have not been comprehensively studied. Therefore, detailed analyses of humoral factors with negative effects on physiological pregnancy and fetal development are urgently needed. In this study, we showed that secreted cyclophilin A (CypA) could be one of such adverse factors.

CypA (18 kDa) is a ubiquitously expressed isomerase that has intracellular and secretory forms. Cytosolic CypA was first discovered as a ligand for the immunosuppressant cyclosporin A [11]. Intracellular CypA functions as a chaperone [12,13] and is involved in many signaling pathways [11,14]. Secreted CypA is a pro-inflammatory factor and attracts stem cells [15], macrophages [16], granulocytes, and activated T cells [17] to the site of infection, thus forming the site of inflammation. Secreted CypA stimulates the production of Th1 cytokines TNFα and IFNγ by CD4+ T cells [18] and induces expression of TNFα by macrophages [19].

In early pregnancy, the endometrial stromal cells produce high levels of secreted CypA and other pro-inflammatory cytokines that are critical for trophoblast invasion into the endometrium during implantation [20,21]. However, further elevations of maternal serum CypA concentrations in the first trimester are associated with the development of various complications (preeclampsia, gestational hypertension, and gestational diabetes) [22].

A comprehensive analysis of CypA’s effects on gestation and fetal development will deepen our understanding of the nature of human pregnancy complications and allow the development of novel strategies for their prevention and treatment. In studies here, using mouse models in vivo, we studied CypA as the factor affecting physiological pregnancy and normal fetal development. In the generated transgenic models of CypA expression in fetal tissues, overproduction of this protein caused mass embryo resorption, mediating partial or complete litter loss and the development of miscarriage. Administration of recombinant human CypA (rhCypA) to pregnant females during fetal organogenesis had teratogenic effects, inducing fetal brain damage and impairing the development of the skull and limb bones of a fetus. Our findings showed that rhCypA could induce macrophage M1 polarization, thus creating a local pro-inflammatory environment at the maternal–fetal interface with elevated levels of pro-inflammatory cytokines exhibiting embryotoxic and teratogenic effects.

## 2. Results

### 2.1. The Fetal mCypA Overexpression Impaired the Antenatal Development of Transgenic Embryos

We generated transgenic embryos with constitutive and inducible mCypA overexpression to evaluate its effects on the embryos’ development.

Transplantation of zygotes carrying the pUC-mCypA transgene resulted in approximately a 3.0-fold decrease in the recipients’ pregnancy rate and the embryo implantation rate compared to the corresponding parameters for transplantation of non-injected zygotes that didn’t carry the transgene (Supplementary Table S1). No viable transgenic mice with constitutive mCypA overexpression were delivered by day 18.5 post-transplantation (Supplementary Table S1). PCR analysis on day 12.5 post-transplantation revealed a high resorption rate of transgenic embryos, which comprised 66% of all resorptions and over 72% of all transgenic embryos (8 of 11) (Supplementary Table S2). These findings suggested that constitutive fetal mCypA overexpression was embryotoxic.

Next, we developed transgenic mice with inducible mCypA expression in osteoblasts by cross-breeding pUC-STOP-mCypA and Osx-Cre mice [23]. Notably, primary transgenic pUC-STOP-mCypA mice were viable and exhibited no phenotypic or functional disorders compared to wild-type littermates and wild-type mice developed from transplanted non-injected zygotes.

To analyze the antenatal development of Cyp-STOP+Cre+ mice, after mating with Osx-Cre males, pUC-STOP-mCypA females didn’t receive DOX for the whole gestation (DOX^¯^ females). This induced mCypA expression in the earliest stages of Cyp-STOP+Cre+ embryos’ development. Control females received DOX for the entire pregnancy (DOX^+^ females). None of the DOX^¯^ females (*n* = 6) delivered a progeny by 21 dpc. All DOX^+^ females (*n* = 6) successfully gave birth to viable offspring, including pups carrying the double transgene (Supplementary Figure S1A). Transgenic Cyp-STOP+Cre+ mice withdrawn from DOX immediately after birth exhibited no phenotypic and functional disorders compared to their transgenic (Cyp-STOP+ and Cre+) and wild-type sibs during 30 days of observation and overexpressed mCypA in the bone tissue (Supplementary Figure S1B).

Autopsy of DOX^¯^ females on 21 dpc revealed late resorptions (Supplementary Figure S2), probably indicating embryo deaths in earlier stages of their post-implantation development. No anomalies or visible signs of resorption were detected in DOX^−^ females (*n* = 4) on 6.5 dpc compared to DOX^+^ females (*n* = 4) (Supplementary Figure S3A). Analysis of DOX^−^ females on 12.5 dpc (*n* = 8) showed a 1.6-fold decreased number of embryos and a 2.6-fold increased resorption rate per female compared to control DOX^+^ females (*n* = 6) (Supplementary Figure S3B, Supplementary Table S3).

Thus, we assumed that embryos of DOX^¯^ females could successfully pass the implantation period (0.5–5.5 dpc) but die in the period of organogenesis (6.5–11.5 dpc). Our data showed that mCypA overexpression in embryonic tissues during organogenesis had a pronounced embryotoxic effect, which resulted in embryonic deaths.

### 2.2. The High Dose of rhCypA Injected into Pregnant Females during Fetal Organogenesis Provoked External Anatomical Anomalies in Mouse Embryos

As human CypA has no species specificity for mice [13,16], to further study the embryotoxicity of CypA, pregnant female mice were injected with rhCypA. Administration of rhCypA didn’t affect the average pregnancy outcomes studied (Supplementary Figure S4), suggesting that the systemic elevation of maternal CypA levels didn’t exhibit embryotoxic effects on developing fetuses.

To analyze anatomical anomalies, fetuses of females in the control (PBS) and rhCypA groups were visually examined (Supplementary Materials). Several fetuses (4%) of control females (PBS) had an asymmetric head (Table 1). The same anatomical anomaly was observed in a few fetuses of females injected with rhCypA during the pre-implantation period or fetogenesis (Table 1). All females dosed with rhCypA during organogenesis delivered fetuses with external and skeletal anomalies. The rate of defective fetuses in this experimental group ranged from 28.6 to 66.6% in individual litters (Supplementary Figure S5A). Notably, in the litter with the maximal rate of defective embryos (litter #7), the mean weight and the mean craniocaudal dimension of fetuses were significantly decreased compared to the control group (PBS) (Supplementary Figure S5A).

**Figure 1:**
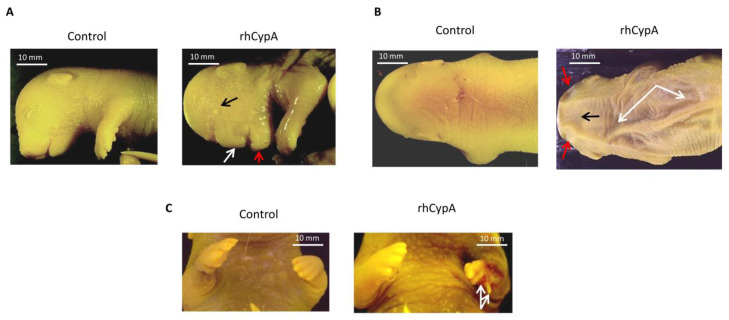
Development of anatomical anomalies in fetuses after systemic injection of rhCypA into pregnant females in the period of organogenesis. Female F1(CBA/Lac × C57BL/6) mice were mated with F1(CBA/Lac × C57BL/6) males. The next morning, females were selected by a copulative plug and s.c. injected with 2.0 mg/mice rhCypA during the period of organogenesis (6.5–11.5 dpc). Control mice received PBS for the whole gestation period. Females were sacrificed on 18.5 dpc, and the anatomical anomalies of the fetuses were visually examined. (**A**) Anatomical anomalies in the facial region of the embryo’s head: an underdeveloped eye (black arrow), defective maxilla and nose (white arrow), and impaired development of the mandible (red arrow). (**B**) Anatomical anomalies in the cranial region of the embryo’s head: deformations of the skull in the zone of the parietal bone (red arrows) and the interparietal bone (black arrow); excessive skin folds on the embryo’s back (white arrows). (**C**) Defects in the distal region of the forelimb: anomalies in the development of the fourth and fifth metacarpals and corresponding phalanges (white arrows).

**Figure 2:**
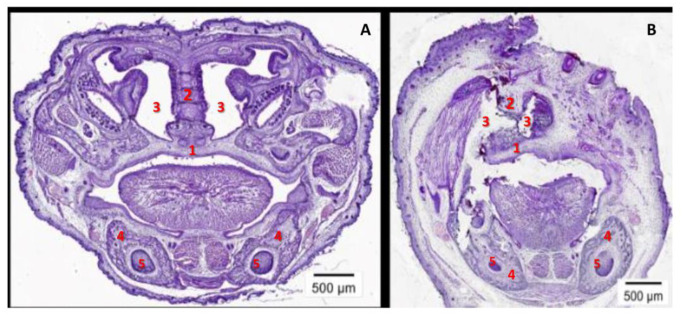
Morphohistological analysis of the facial region of the embryo’s head. The frontal section through the visceral region of the skull at the level of the nasal cavity. (**A**) A fetus of a control female (PBS): the normal development of the hard palate (1), the nasal bone (2), the nasal cavity (3), mandibular bones (4), and tooth germs (5). (**B**) A fetus of a female rhCypA-injected during organogenesis (6.5–11.5 dpc): marked asymmetry of the visceral region, impaired formation of the hard palate (1) and the nasal bone (2), an asymmetric nasal cavity (3); the development of the lower jaw, including osteogenesis of the mandibular bones (4), and the development of tooth germs (5) are not affected.

Females injected with rhCypA during organogenesis delivered pups (16 of 69—23.0%; Table 1) with severe anatomical anomalies—multiple defects in the facial (Figure 1A) and cranial (Figure 1B) regions of the head. Several fetuses (7 of 69—4.8%) of these females had significant eye position asymmetry and marked underdevelopment of one eye and eyelids (Figure 1A, black arrow). The developmental stage of defective eyes corresponded to stages 21–22 of normal mouse embryonic development [24]. These fetuses had defective upper jaws and noses (Figure 1A, white arrow). Morphohistological analysis showed a significant asymmetry of the visceral region, the defective hard palate and nasal bone, and the asymmetric nasal cavity in the fetuses of these rhCypA-dosed females (Figure 2B, 1–3). The mandibles of these embryos were less defective (Figure 1A, red arrow), and the formation of mandibular bones and tooth germs was not impaired (Figure 2B, 4, 5).

Embryos of females rhCypA-dosed during organogenesis also had skull deformations in the region of the parietal (Figure 1B, red arrows) and interparietal bones (Figure 1B, black arrows) and an excessive skin fold on their back (Figure 1B, white arrows), possibly indicative of impaired derma development. Of the defective fetuses, 12.5% (2 of 16) developed anomalies in the posterior region of the autopod (4–5 metacarpals and corresponding phalanges) (Figure 1C, white arrows). The histological analysis of the deformed forelimb (Figure 1C) revealed the absence of the fourth metacarpal (Supplementary Figure S6, 4*) and a distinct anomaly of the fifth metacarpal (Supplementary Figure S6, 5*).

### 2.3. The High Level of Maternal CypA during Organogenesis Induced Skeletal Defects in Mouse Embryos

Defects in the embryos’ development described above indicated that rhCypA could probably be a teratogen, which impaired bone formation in a fetus. Therefore, we analyzed the skeletons of fetuses of control and rhCypA-dosed females (Supplementary Materials). Fetuses of control (PBS) females and females injected with rhCypA during pre-implantation had symmetric ossification of all bones without developmental defects (Table 1). Administration of rhCypA to pregnant females during fetogenesis resulted in impaired skeletal formation only in two fetuses (Table 1).

**Figure 3:**
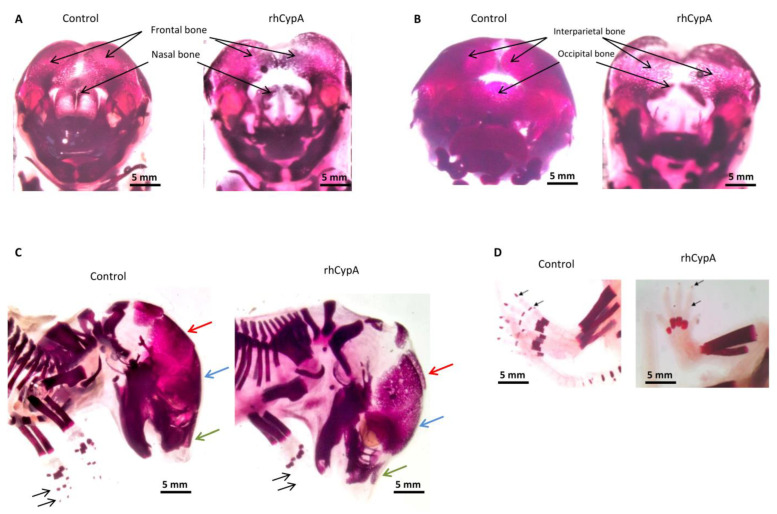
Skeletal anomalies in fetuses after systemic administration of rhCypA to pregnant females in the period of organogenesis. Female F1 (CBA/Lac × C57BL/6) mice were mated with male F1(CBA/Lac × C57BL/6) mice. The next morning, females were selected by a copulative plug and s.c. injected with 2.0 mg/mice rhCypA within the period of organogenesis (6.5–11.5 dpc). Control mice received PBS for the whole gestation period. Females were sacrificed on 18.5 dpc, fetuses were stained with alizarin red, and skeletal anomalies were visually examined. (**A**) Impaired ossification and development of the frontal and nasal bones. (**B**) Anomalies in the development of the interparietal and occipital bones. (**C**) Defects in ossification and development of the parietal bones (red arrow). Defective ossification of the frontal (blue arrow) and nasal (green arrow) bones. Absence of distal phalanges on the forelimbs (black arrows). (**D**) Absence of distal phalanges on the hind limb (black arrows).

We found significant abnormalities in the ossification and development of the skull bones in 7 of 30 fetuses (23.0%) born by females injected with rhCypA during organogenesis (Table 1). Four of these fetuses had underdevelopment of the single mandible and/or maxilla, a lack of fusion, or asymmetric fusion of the mandibles or maxillae. Moreover, some of these embryos had defects in the ossification and development of the frontal bone (Figure 3A,C, blue arrow), with distinct deformation of the medial zones (Figure 3A) and the nasal bones (Figure 3A,C, green arrow). We also observed a marked delay in the ossification and development of the interparietal and occipital bones (Figure 3B). Furthermore, the anterior and medial zones of the parietal bone were significantly deformed and underdeveloped (Figure 3C, red arrow).

In the skeleton of three embryos (10%) of females rhCypA-dosed during organogenesis, we detected impaired ossification of distal phalanges on the forelimbs (Figure 3C) and hind limbs (Figure 3D) or no ossification of the carpus and metacarpus on both limbs. This observation further confirmed the teratogenic effects of rhCypA injected into pregnant females during organogenesis.

Altogether, our studies showed that elevation of CypA levels in the organism of a pregnant female is especially dangerous to a fetus in the period 6.5–11.5 dpc and can provoke developmental anomalies. High maternal CypA concentrations during organogenesis can induce skeletal defects, mainly in the visceral and cranial regions of the skull and distal limb bones of mouse fetuses.

### 2.4. The High Level of Maternal CypA during Organogenesis Damaged the Fetal Brain’s Development

Morphogenesis of the brain precedes chondro- and osteogenesis. The facial skeleton largely consists of the neural crest cell derivatives that migrate to form the facial primordia [25,26,27]. Therefore, brain growth influences facial development [26], and anomalies in the facial morphogenesis observed in fetuses of females injected with rhCypA during organogenesis (Figure 3) could be due to impairment of the embryos’ brain development [25].

**Figure 4:**
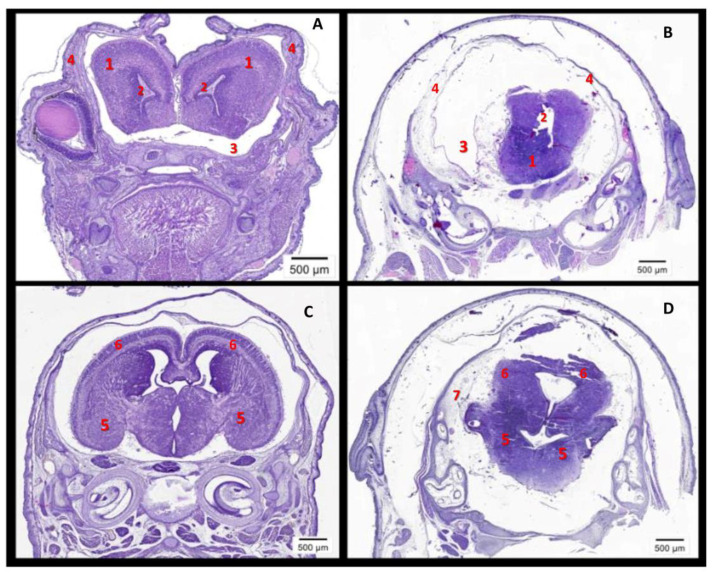
Defective brain development in fetuses after systemic rhCypA injection into pregnant females in the period of organogenesis. F1(CBA/Lac × C57BL/6) females with a copulative plug were s.c. injected with 2.0 mg/mice rhCypA within the period of organogenesis (6.5–11.5 dpc). Control mice received PBS for the whole gestation period. Females were sacrificed on 18.5 dpc, and the histological analysis of the fetal brain was performed. (**A**,**B**) The frontal section at the level of olfactory bulbs. (**A**) A fetus of a control female (PBS): normal development of olfactory bulbs (1) and lateral ventricles (2). (**B**) A fetus of a rhCypA-dosed female: defects in the brain development, which resulted in the underdevelopment of olfactory bulbs (1) and lateral ventricles (2); severe hydrocephalus due to the excessive accumulation of the cerebrospinal fluid in lateral ventricles (2) and subarachnoid spaces (3); the defective formation of the cranial region of the skull (4). (**C**,**D**) The frontal section at the level of the cerebral hemispheres. (**C**) A fetus of a control female (PBS): normal development of cerebral hemispheres (5) and the cerebral cortex (6). (**D**) A fetus of a rhCypA-dosed female: defective formation of cerebral hemispheres (5), absence of the formed cerebral cortex (6), and severe hydrocephalus (7).

Histological evaluations revealed severe brain defects in the embryos of rhCypA-dosed females (Figure 4). Compared to embryos delivered by control females (PBS) (Figure 4A,C), in the brain of these fetuses, the olfactory bulbs and lateral ventricles of the cerebral hemispheres were underdeveloped (Figure 4B, 1–2), and the formation of the cerebral hemispheres and the cerebral cortex was significantly impaired (Figure 4D, 5–6). Moreover, severe hydrocephalus was developed due to the excessive accumulation of cerebrospinal fluid in the lateral ventricles and subarachnoid spaces (Figure 4B, 3; Figure 4D, 7). It was accompanied by the defective formation of the cranial region of the skull (Figure 4B, 4) and increasing atrophy of brain tissues (Figure 4B,D). In clinical practice, such multiple brain anomalies usually result in neonatal deaths or stillbirths. Thus, our studies showed that rhCypA effects on embryos during organogenesis led to impaired development of their brains and, consequently, defects in the facial region of their skulls.

### 2.5. RhCypA Induced Macrophage M1 Polarization In Vitro via Activation of STAT1 and STAT3 Signaling Pathways

The immune microenvironment at the maternal–fetal interface plays a crucial role in the establishment and development of pregnancy. In particular, the prevalence of M2 macrophages in the decidua is essential for normal pregnancy, while the accumulation of M1 macrophages is associated with pregnancy complications [28,29].

The previous study exploited the THP-1 in vitro model to unveil the link between the M1/M2 polarization bias and various pregnancy pathologies, including preeclampsia [30]. In the studies here, we used the same in vitro model to reveal possible mechanisms of rhCypA embryotoxic and teratogenic activity by evaluating its effects on macrophage M1/M2 polarization. For this, THP-1 cells were cultured in vitro with increasing doses of rhCypA, ranging from physiological concentrations [30] to the levels reached under different pathological conditions [19,30].

**Figure 5:**
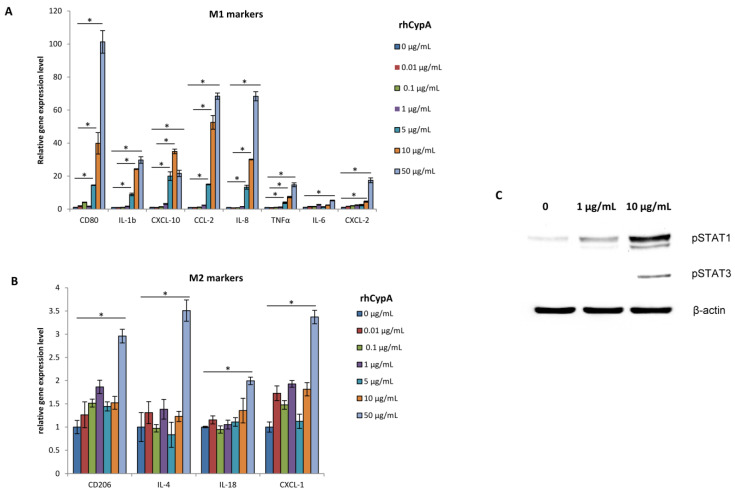
Effects of rhCypA on macrophage M1/M2 polarization. THP-1 cells were cultured with rhCypA at the indicated concentrations for 24 h. Relative gene expression levels of M1 (**A**) and M2 (**B**) markers were evaluated by qPCR. The data of one representative experiment is presented as mean ± SD for three technical repeats. (**C**) Expression levels of phosphorylated STAT1 and STAT3 were analyzed by Western blot. * *p* < 0.01 (Mann–Whitney U-test).

Analysis of M1/M2 macrophage gene signatures showed that in doses approximated to its physiological levels (0.01–1 μg/mL), rhCypA didn’t affect the gene expression of both M1 and M2 markers (Figure 5A,B). But, starting with 5 μg/mL, rhCypA significantly stimulated the expression of all analyzed M1 genes in a dose-dependent manner (Figure 5A). At all but the highest dose, rhCypA had no effects on M2 gene expression in THP-1 cells (Figure 5B). At 50 μg/mL, it stimulated the expression of both M1 and M2 genes, but the relative gene expression of M2 markers was markedly lower compared to the levels of M1 gene expression induced by this dose of rhCypA (Figure 5A,B). Notably, the rhCypA dose of 50 μg/mL is a non-physiological concentration that is never seen, even under pathological conditions associated with inflammation [19,30].

To identify signaling pathways involved in rhCypA-mediated positive regulation of M1 gene expression, the phosphorylation levels of STAT1 and STAT3 in THP-1 cells were evaluated by Western blot (Figure 5C). STAT1 phosphorylation levels were significantly increased under 1 μg/mL and 10 μg/mL rhCypA, with a distinct dose dependence (Figure 5C). Interestingly, STAT3 phosphorylation level was increased in THP-1 cells only after co-culture with 10 μg/mL rhCypA (Figure 5C), the dose strongly inducing M1 gene expression (Figure 5A). These findings indicated that rhCypA positively regulated STAT1 and STAT3 phosphorylation, with STAT1 being more sensitive to its action. Furthermore, our findings revealed that up-regulation of M1 genes required simultaneous activation of both the STAT1 and STAT3 signaling pathways in THP-1 cells, which could only be triggered by increased rhCypA levels.

## 3. Discussion

In the studies here, we created transgenic mice with constitutive and inducible mCypA expression to directly analyze the effects of its increased production in a fetus. Moreover, we evaluated the embryotoxic and teratogenic effects of rhCypA under its systemic administration to pregnant mice.

In the model of mCypA constitutive expression, we detected the low pregnancy rate of recipient females on 12.5 dpc and 18.5 dpc, which could indicate the loss of all embryos by these terms. In DOX¯ females on 12.5 dpc, their litter size was significantly reduced, and their resorption rate was considerably higher compared to DOX^+^ females whose embryos didn’t express mCypA. Notably, no pregnancy pathologies were observed in DOX^¯^ females on 6.5 dpc, indicating that their embryos could successfully pass the implantation period (0.5–5.5 dpc).

However, these effects could depend on the onset of mCypA expression in transgenic embryos. To generate transgenic mice, we used the pUC18–Col2.3 expression vector that drove the transgene expression in osteoblasts [31]. The 2.3 kb rCo1a1 promoter activity was detected in the forming bone tissue starting at E14.5 [31]. However, we observed mass embryo resorptions, predominantly transgenic (over 60%; Supplementary Table S2), by 12.5 dpc, suggesting that mCypA expression could be induced in osteoblast progenitors in the earlier stages of the transgenic embryos’ development. Early mesodermal precursors with the potential to differentiate into mesenchymal lineage cells (bone and cartilage) were identified in mouse embryos on E9.5, and mesenchymal progenitors capable of differentiation into osteogenic lineage cells were detected at 11 dpc [32,33]. Experiments in vitro demonstrated osteoblast differentiation from mouse embryonic stem cells by day 7 of culturing [34]. Thus, our expression vectors could not drive the transgene expression during 0.5–5.5 dpc; therefore, it was impossible to directly evaluate the effects of mCypA overexpression in transgenic embryos on their implantation. 

mCypA could exhibit embryotoxic effects during fetal organogenesis since 8.5–10.5 dpc is the critical gestational period, during which the morphologically and functionally mature placenta forms and provides sufficient maternal–fetal exchange [35]. High levels of CypA, similar to other pro-inflammatory cytokines, could impair placentation, leading to defects in fetal development and fetal death. This effect of CypA could be mediated by inducing the expression of serum amyloid alpha (SAA) [36]. SAA at significantly elevated concentrations inhibits trophoblast invasiveness [37] and contributes to early pregnancy loss [38]. Similarly, it was shown that increased concentrations of IFNγ worsen placental functions by inhibiting trophoblast invasiveness, impairing angiogenesis, and inducing vascular dysfunction [39,40]. Enhanced secretion of TNFα promotes apoptosis of the trophoblast and suppresses its invasion [41,42,43], and its elevated local concentrations induce thrombosis and placental ischemia [41].

The predominance of anti-inflammatory cytokines in the uterus is necessary in the period of organogenesis to induce and maintain maternal–fetal tolerance. It seems plausible that CypA overexpressed in transgenic fetuses could shift the cytokine balance at the maternal–fetal interface towards pro-inflammatory cytokines, resulting in considerably increased rates of abortion and embryo resorption in females carrying fetuses with constitutive or inducible mCypA expression.

Our data suggest that, as a pro-inflammatory factor, CypA overexpressed in transgenic embryos could induce immune-mediated fetal death. Previous studies demonstrated that the increased fetal secretion of pro-inflammatory mediators (TNFα, IL-6, etc.) induced by intrauterine infection or sterile inflammation [44,45,46] could activate the maternal immune system, enhancing the immune cells trafficking into the fetus, stimulating the accumulation and activation of effector cells in the uterus, and increasing the local production of embryotoxic cytokines (e.g., IFNγ) [44,47]. As the chemoattractant for activated cells of the innate and adaptive immunity and the regulator of the production of pro-inflammatory cytokines by activated T cells and macrophages [16,17,18,19], CypA expressed in fetal tissues could promote a condition similar to fetal inflammatory response syndrome (FIRS) characterized by the loss of maternal–fetal tolerance, the development of multi-organ failure in the fetus, pre-term membrane rapture, and pre-term labor [44,45].

To date, the increased level of pro-inflammatory cytokines (particularly IL-1β and IL-6) is used as a marker of intrauterine infection and FIRS [46,48]. Our data allow us to confirm CypA as another prognostic factor [22] that could be applied for the earliest diagnosis of different pregnancy complications. The role of CypA in the development of complicated pregnancy was indirectly demonstrated by the effects of cyclosporin A as the ligand for CypA on the improvement of pregnancy outcomes in abortion-prone mice [49,50].

Our in vivo models of rhCypA systemic administration to pregnant female mice could reproduce possible complications of human pregnancy associated with inflammatory processes in the mother’s organism. Injected during the period of organogenesis and active formation of the placenta (8.5–10.5 dpc) [35], rhCypA caused dramatic teratogenic effects leading to severe fetal brain pathologies, usually fatal in clinical practice. Fetuses of rhCypA-dosed females developed anencephaly, probably resulting from impairment of neuraxis formation early in pregnancy. It was shown that persistent viral infections in the maternal organism could cause potent teratogenic effects and provoke such developmental defects in humans [51]. The cytokine shift towards pro-inflammatory factors altered normal fetal brain development, resulting in postnatal behavioral and cognitive disorders in the offspring [52,53,54,55].

Furthermore, in the fetuses of these rhCypA-dosed females, we observed impaired ossification and development of the skull and distal limb bones that led to the formation of multiple anatomical and skeletal anomalies. Our data correlate with the previous studies demonstrating that increased levels of pro-inflammatory cytokines (IL-1, TNFα, IL-6) are associated with defective bone formation by enhancing apoptosis in osteoblasts, decreasing bone mineral density, and stimulating bone resorption [56,57,58,59,60]. Notably, the described anomalies of the facial skull could be due to either direct rhCypA effects on the bone development and ossification or impairment of the brain vesicle formation, as the brain development influences the facial skull formation. Since both of these processes depend on neural crest development [25], it seems possible that CypA at high levels could impair the formation of neural crest derivatives.

The precise balance of immune cells and cytokines at the maternal–fetal interface is essential for the establishment and maintenance of a normal pregnancy [1,2]. As the second largest cell type in the decidua [61], macrophages contribute greatly to pregnancy processes [28]. The prevalence of immunosuppressive M2 macrophages is required for sustaining maternal–fetal tolerance and the development of a normal pregnancy [28]. The bias towards pro-inflammatory M1 decidual macrophages is linked to defective trophoblast invasion and impaired remodeling of uterine vessels, resulting in preeclampsia, spontaneous abortion, or preterm birth [28,29,62].

In this study, we found that high doses of rhCypA induced M1 macrophage polarization via activation of the STAT1/3 signaling pathways (Figure 5). Our findings are in line with previous studies that showed that pro-inflammatory cytokines regulate activation of the STAT1/3 pathway, promoting M1 polarization [63,64]. Therefore, we propose that increased levels of maternal CypA could shift a balance of polarization between M2 and M1 macrophages towards M1 macrophages at the maternal–fetal interface, thus promoting the establishment of a local pro-inflammatory microenvironment and elevating the levels of pro-inflammatory cytokines shown to be embryotoxic and teratogenic [6,7,8,9,10].

This study directly demonstrated the toxicity of high CypA levels for embryos during their organogenesis, both when it is overexpressed in fetal tissues or artificially increased in the organism of a pregnant female. Our results indicated CypA as a pro-inflammatory factor that could influence the balance of intrauterine cytokines and M1/M2 macrophages, regulate fetal development, and mediate severe pregnancy complications. Thus, CypA could be used as a marker of complicated pregnancy and as a therapeutic target for the correction of pregnancy complications.

## 4. Material and Methods

Mice. Female and male C57BL/6 and F1(CBA/Lac × C57BL/6) mice were obtained from the breeding facility of the N.N. Blokhin National Medical Research Center of Oncology of the Ministry of Health of the Russian Federation (N.N. Blokhin NMRCO, Moscow, Russia). Males (18–22 g) and females (12–13 g) were used to obtain fertilized zygotes for microinjections. Hybrid F1(CBA/Lac × C57BL/6) females (23–24 g) mated with vasectomized males (18–22 g) were used as recipients [65,66,67]. Transgenic Osx-Cre mice (on the C57BL/6 background) [23] with the tetracycline-dependent expression (tet-off) of Cre-recombinase in osteoblasts were kindly provided by the Department of Clinical Pathobiochemistry, Institute for Clinical Chemistry and Laboratory Medicine, Technische Universität (Dresden, Germany). Mice were housed in facilities maintained at 20–24 °C with a 40% relative humidity and a 12-h light/dark cycle. All mice had ad libitum access to standard rodent chow and filtered tap water. Experimental groups consisted of 3–11 animals. Mice were handled in strict compliance with the NIH guide for the care and use of laboratory animals (8th edition, 2011). The protocol was approved by the Committee on the Ethics of Animal Experiments of N.N. Blokhin NMRCO and the Institute of Gene Biology of the Russian Academy of Sciences (Moscow, Russia). 

Development of transgene DNA constructions. Total RNA from thymocytes of a C57BL/6 mouse was isolated using TRI-reagent (MRC, Inc, Cincinnati, OH, USA) followed by cDNA synthesis using the RevertAid First Strand cDNA Synthesis Kit (Thermo Scientific, Waltham, MA, USA). The full-length gene of murine CypA (mCypA) was amplified using the specific primers (Supplementary Table S4) and cloned into the pUC18–Col2.3 cassette vector (kindly provided by Mark Kronenberg, Department of Reconstructive Sciences, University of Connecticut Health Center, USA) by NheI and ClaI restriction sites. The pUC18–Col2.3 cassette vector provided local overexpression of the transgene in osteoblasts [68,69]. The transgene sequence pUC-mCypA was cut from the plasmid backbone by SalI restriction. To generate the pUC-STOP-mCypA expression vector, a ~3.9 bp fragment contained the 5′-loxP site and nearly 2/3 of the STOP-cassette was cut from the LoxP-STOP-LoxP TOPO plasmid (#11584, Addgene, Watertown, MA, USA) by BamHI and cloned by blunt ends into the pUC-mCypA expression construct digested by EcoRV (pUC-5′STOP-mCypA). We could not clone the entire STOP-cassette as its 3′- part and the 3′-loxP site contained EcoRV restriction sites. To clone the remaining 3′- part of the STOP-cassette and the 3′-loxP site, the LoxP-STOP-LoxP TOPO plasmid was cut by XhoI and ApaI and cloned into the pT2/HB plasmid (#26557, Addgene). The 3′- part of the STOP-cassette and the 3′-loxP site were cut from the resulting plasmid by BamHI and cloned into pUC-5′STOP-mCypA. The transgene sequence pUC-STOP-mCypA was obtained by KpnI restriction and SacII partial restriction (Supplementary Figure S7).

Generation of transgenic mice. Primary transgenic mice were generated by DNA microinjection into the pronucleus of fertilized eggs, as described elsewhere [65,66]. After injection, zygotes were cultured as described earlier [67] for 2 h at 37 °C and 5.0% CO_2_. Subsequently, zygotes with no visible signs of damage were considered viable and transferred into the oviduct of pseudopregnant recipient females [67]. The genotyping of progeny was performed by PCR using specific primers (Supplementary Table S4). To establish the pUC-STOP-mCypA line, primary transgenic mice generated on the F1(CBA/Lac × C57BL/6) background were backcrossed with C57BL/6 mice for 6 generations. To generate transgenic mice with inducible mCypA overexpression, pUC-STOP-mCypA mice were mated with Osx-Cre mice. To prevent mCypA expression in the antenatal period of the embryo’s development, animals in this breeding constantly received doxycycline (DOX, 1.0 mg/mL) with water ad libitum. To induce mCypA expression in Cyp-STOP+Cre+ mice, DOX was withdrawn immediately after delivery, and Cyp-STOP+Cre+ mice were constantly kept without DOX.

Recombinant human cyclophilin A (rhCypA), schemes of administration. RhCypA (E. coli) was produced as previously described [36,70]. F1(CBA/Lac × C57BL/6) female and male mice (18–22 g) were mated, and females were selected the next morning by a copulative plug (0.5 day post-coitus, dpc). These females were randomly divided into 3 groups and subcutaneously (s.c.) injected with 2.0 mg/mouse rhCypA in 200.0 μL PBS as follows: (1) 0.5–5.5 dpc (pre-implantation period) (n = 7); (2) 6.5–11.5 dpc (organogenesis) (n = 9); or (3) 12.5–17.5 dpc (fetogenesis) (n = 8). This rhCypA dose was chosen as the maximally tolerated dose without toxic effects under the course administration according to our preliminary studies [36]. Control females (n = 7) similarly received PBS for the whole gestation.

Evaluation of rhCypA embryotoxic effects. All experimental females were sacrificed by cervical dislocation on 18.5 dpc, and their uterine horns were isolated. The numbers of implantation sites, live and resorbed embryos were counted. Each embryo was dissected from the uterus and weighed; its craniocaudal dimension was also measured. A litter of each female was divided into two groups: nearly 2/3 of embryos were fixed in Bouin’s fixative (LabChem, Zelienople, PA, USA), and 1/3 of embryos were fixed in 96° ethanol for at least 7 days. Fetuses fixed in Bouin’s fixative were subsequently visually analyzed using a Zeiss Stemi Dv4 stereomicroscope (Carl Zeiss, Oberkochen, Germany) (Supplementary Materials). To analyze skeletal development, after fixation in ethanol, embryos were stained with alizarin red (Interhim, Moscow, Russia) as described elsewhere [71]. The ossification of embryos’ skeleton was examined using a stereomicroscope Olympus SZ 61 (Olympus, Tokyo, Japan).

Histological analysis of embryos. Embryos fixed in Bouin’s fixative for 7 days were dehydrated in a gradient of alcohols and embedded in Histomix (BioVitrum, St. Petersburg, Russia). Frontal tissue sections (5 μm) of the embryos’ head and dorsal tissue sections (5 μm) of the embryos’ limbs were prepared using an AccuCut SRM 200 microtome (Sakura, Alphen den Rijn, The Netherlands) and stained with hematoxylin-eosin (Gemstandart, St. Petersburg, Russia). All samples were examined using a slide scanner Olympus VS120 based on a microscope Olympus BX-61VS (Olympus). Histological evaluations were performed blinded to the animal treatment.

Cell line, culture conditions. The human monocyte cell line THP-1 (TIB-202, ATCC) was cultured in RPMI-1640 medium (PanEco, Moscow, Russia) supplemented with 10% fetal bovine serum (HyClone, GE Healthcare, Chicago, IL, USA), 0.01 mg/mL ciprofloxacin (KRKA, Novo Mesto, Slovenia), and 0.01 M HEPES (PanEco) (complete RPMI). Cells at 70% confluence were harvested, counted in a hemocytometer after trypan blue/eosin (mixed 1:1 *v*/*v*) staining, and seeded at 5 × 10^5^ cells/mL in 6-well plates (Corning Costar, Sigma Aldrich, St. Louis, MO, USA) in 2 mL of supplemented RPMI. RhCypA was added to cells at doses of 0.01, 0.1, 1, 5, 10, and 50 μg/mL in 100 μL of complete RPMI. Cells were then cultured at 37 °C with 5% CO_2_ for 24 h, collected, and washed in PBS by centrifugation (200× *g*, 5 min, 4 °C). Total RNA from THP-1 cells was then isolated, followed by cDNA synthesis as described above, and then proceeded to qPCR analysis. Alternatively, for western blot analysis, THP-1 cells cultured as described above were lysed in RIPA buffer supplemented with a protein inhibitor cocktail (Sigma Aldrich).

Real-time PCR (qPCR). Femurs, the bone marrow, and the spleen of wild-type, Cyp-STOP+, and Cyp-STOP+Cre+ littermate mice kept without DOX (1-mo-of-age) were isolated and powdered in liquid nitrogen. Total RNA isolation and cDNA synthesis were performed as described above. The specific primers for mouse *Ppia* were used: 3′-GACTGAATGGCTGGATGG-5′ and 3′-CAGAAGGAATGGTTTGATGG-5′; *hprt* and *tbp* were used as housekeeping genes. Primers used for qPCR analysis of the M1/M2 gene expression profiles in THP-1 cells are listed in Supplementary Table S5. *CAP1* was used as a housekeeping gene. qPCR was performed on BioRad CFX96 (Hercules, CA, USA) using SYBR Green (DNK-Syntez, Moscow, Russia). Results were analyzed using the CFX Manager Software (BioRad). Relative gene expression levels were calculated using the ΔΔCt method.

Western blot. The concentration of total protein isolated from THP-1 cells as described above was quantified by the Bradford method. Absorbance at 560 nm was measured with a CLARIOstar Plate Reader (BMG Labtech, Ortenberg, Germany). Proteins were separated by SDS-PAGE and transferred onto a 0.2 μm nitrocellulose membrane (BioRad). After blocking with 5% skimmed milk, membranes were treated with primary antibodies and incubated at 4 °C overnight. Membranes were washed with TBS-T and incubated for 1 h at room temperature with secondary antibodies (#7074 and #7076, Cell Signaling, Danvers, MA, USA). Membranes were visualized with the Clarity Western ECL Substrate or ClarityMax Western ECL Substrate (BioRad) using iBright FL1500 Imaging System (Invitrogen, Waltham, MA, USA). The following primary antibodies were used: anti-pSTAT1 (D4A7) (#7649, Cell Signaling) and anti-pSTAT3 (D3A7) (#9145, Cell Signaling). β-actin (AC004, ABclonal, Woburn, MA, USA) was used as a housekeeping protein.

Statistical analysis. Data are presented as mean ± SD. All statistical analyses were performed using the Mann–Whitney U-test using the Prism software (v. 8.1.2, GraphPad, San Diego, CA, USA). A *p*-value < 0.05 was considered significant.

## Figures and Tables

**Table 1:** Analyses of the embryotoxic effect of rhCypA systemic administration to pregnant female F1(CBA × C57BL/6) mice.

Parameter		Control (PBS)	rhCypA 2.0 mg/mouse		
			0.5–5.5 dpc (Pre-Implantation Period)	6.5–11.5 dpc (Organogenesis)	12.5–17.5 dpc (Fetogenesis)
**Analysis of anatomical anomalies**					
Total number of fetuses studied	49		55	69	56
with anatomical anomalies (%)	2 (4.1)		2 (3.6)	**16** (**23.0**)	1 (1.8)
**Analysis of skeletal anomalies**					
Total number of fetuses studied	24		24	30	29
with skeletal anomalies (%)	0		0	**7** (**23.3**)	2 (**6.8**)

## Data Availability

Not applicable.

## Supplementary Materials

The following supporting information can be downloaded at: https://www.mdpi.com/article/10.3390/ijms241411279/s1.

## Abbreviations

NK—natural killers, Th—T helper cells, CypA—cyclophilin A, rhCypA—recombinant human CypA, dpc—day post-coitus, DOX—doxycycline.
