# Supplementary material for: Cyclophilin A as a Pro-Inflammatory Factor Exhibits Embryotoxic and Teratogenic Effects during Fetal Organogenesis
Source: Int J Mol Sci. 2023 Jul 10;24(14):11279. doi: 10.3390/ijms241411279 (PMC10380070; doi:10.3390/ijms241411279)
Supplement: Supplementary file 1 [file ijms-24-11279-s001.zip › Supplementary Table S1.pdf]

Supplementary Table S1. The efficiency of generation of transgenic mice pUC-mCypA with the constitutive overexpression of mCypA evaluated on Day 18.5 post- zygotes transplantation

Zygotes donors	Number of transplanted embryos	Pregnancy rate of recipients[1] (%)	Embryos transplantation rate[2] (%)	Number of pups per 1 female	Transgenesis efficiency[3] (%)	Number of live transgenic pups[4]
C57BL/6 microinjected zygotes	237	4/29 (13.8)	12/237 (5.0)	3.0	2/12 (16.7)	0/2
F1(CBA x C57BL/6) microinjected zygotes	390	14 + 1 abortion/50 (30.0)	26/390 (6.7)	1.7	1/26 (3.8)	0/1
**Total** for microinjected zygotes	627	19/79 (24.0)	38/627 (6.1)	2.3	3/38 (7.9)	0/3
F1(CBA x C57BL/6) intact (non-microinjected) zygotes	100	7/10 (70.0)	18/100 (18.0)	2.5	-	-

[1] Number of pregnant recipients / Total number of recipients

[2] Number of newborns / Total number of transplanted embryos

[3] Number of transgenic pups / Total number of newborns

[4] Number of live transgenic pups / Total number of transgenic pups
