# Supplementary material for: Cyclophilin A as a Pro-Inflammatory Factor Exhibits Embryotoxic and Teratogenic Effects during Fetal Organogenesis
Source: Int J Mol Sci. 2023 Jul 10;24(14):11279. doi: 10.3390/ijms241411279 (PMC10380070; doi:10.3390/ijms241411279)
Supplement: Supplementary file 1 [file ijms-24-11279-s001.zip › Supplementary Table S2.pdf]

Supplementary Table S2. The survival rate of transgenic pUC-mCypA embryos on Day 12.5 post - transplantation

Type of embryos	Total number of embryos	Number of transgenic embryos (%)
Live	10	3 (30.0)
Resorbed	12	8 (66.7)
TOTAL	22	11 (50.0)
