# Supplementary material for: Cyclophilin A as a Pro-Inflammatory Factor Exhibits Embryotoxic and Teratogenic Effects during Fetal Organogenesis
Source: Int J Mol Sci. 2023 Jul 10;24(14):11279. doi: 10.3390/ijms241411279 (PMC10380070; doi:10.3390/ijms241411279)
Supplement: Supplementary file 1 [file ijms-24-11279-s001.zip › Supplementary Table S3.pdf]

Supplementary Table S3. Analysis of females from the breeding pUC-STOP-mCypA x Osx-Cre on Day 12.5 post-coitus

Group	Total number of females examined	Total number of embryos	Number of embryos per 1 female	Rate of embryos resorption, %
DOX^{-}	8	28	3.5 ± 1.3	20.3 ± 13.0
DOX^{+}	6	35	5.8 ± 0.8	7.8 ± 3.7
