# Supplementary material for: Cyclophilin A as a Pro-Inflammatory Factor Exhibits Embryotoxic and Teratogenic Effects during Fetal Organogenesis
Source: Int J Mol Sci. 2023 Jul 10;24(14):11279. doi: 10.3390/ijms241411279 (PMC10380070; doi:10.3390/ijms241411279)
Supplement: Supplementary file 1 [file ijms-24-11279-s001.zip › Supplementary Table S4.pdf]

Supplementary Table S4. Primers used for mCypA cloning and PCR genotyping of transgenic mice pUC-mCypA, pUC-STOP-mCypA, and Osx-Cre

Gene/Mice line	Forward primer (5`→3`)	Reverse primer (5`→3`)
mCypA (cloning)*	ATC**GCTAGC**ATGGTCAACCCCACCGTGTTC	CG**ATCGATCTA**CAGAAGGAATTGTATGAT
pUC-mCypA	GGAACCCACTGCCCACAACTCCTG	CGATCGATCTACAGAAGGAATTGTATGAT
pUC-STOP-mCypA	GCCTGAAGAACGAGATCAGC	GCAAACAGCTCGAAGGAGAC
Osx-Cre	GCGGTCTGGCAGTAAAAACTATC	GTGAAACAGCATTGCTGTCACTT
TAP**	TGCCTAAGAAGCTGGGAAAAGT	AGACTTCAGCCACGTAAGCCAA

*NheI and ClaI restriction sites, contained in the forward and reverse primer, respectively, are marked in bold

** used as a housekeeping gene
