# Supplementary material for: Cyclophilin A as a Pro-Inflammatory Factor Exhibits Embryotoxic and Teratogenic Effects during Fetal Organogenesis
Source: Int J Mol Sci. 2023 Jul 10;24(14):11279. doi: 10.3390/ijms241411279 (PMC10380070; doi:10.3390/ijms241411279)
Supplement: Supplementary file 1 [file ijms-24-11279-s001.zip › Supplementary Table S5.pdf]

Supplementary Table S5. Primers used for qPCR analysis of M1 and M2 gene expression profile in THP-1 cells

Gene	Forward primer (5`→3`)	Reverse primer (5`→3`)
IL1b	TCGCCAGTGAAATGATGGCT	TGGAAGGAGCACTTCATCTGTT
TNFa	CACTTTGGAGTGATCGGCCC	TTGTCACTCGGGGTTCGAGA
CXCL1	CTGCCTTACAACAAAGGGGCT	TAAAGGTAGCCCTTGTTTCCCC
CXCL1O	AAGTGGCATTCAAGGAGTACCT	GGACAAAATTGGCTTGCAGGA
CCL2	TCTCAAACTGAAGCTCGCAC	CATTGATTGCATCTGGCTGAG
CXCL2	ACAGTGTGTGTGGTCAACATTTCTC	TCTGCTCTAACAGAGGGAA
IL8	AGTTTTTGAAGAGGGCTGAGAAT	CAACAGACCCACACAATACATGA
IL6	CACAGACAGCCACTCACCTC	TTTTCTGCCAGTGCCTCTTT
CD80	CGCCTCTCTGAAGATTACCCAAA	CCTGGGTCTCCAAAGGTTGTG
CD206	GAGGGATGCTCTGACCACCT	GGGTCCCATCACTCCACTCA
IL-4	ACATCTTTGCTGCCTCCAAGAAC	AGCGAGTGTCCTTCTCATGGT
IL-18	GAAAACCTGGAATCCGATTACTT	R CCATACCTCTAGGCTGGCTATCT
CXCL1	CTGCCTTACAACAAAGGGGCT	TAAAGGTAGCCCTTGTTTCCCC
*CAP1**	ATTCCCTGGATTGTGAAATAGTC	ATTAAAGTCACCGCCTTCTGTAG

*used as a housekeeping gene
