# Supplementary figures and images for: Cyclophilin A as a Pro-Inflammatory Factor Exhibits Embryotoxic and Teratogenic Effects during Fetal Organogenesis
Source: Int J Mol Sci. 2023 Jul 10;24(14):11279. doi: 10.3390/ijms241411279 (PMC10380070; doi:10.3390/ijms241411279)

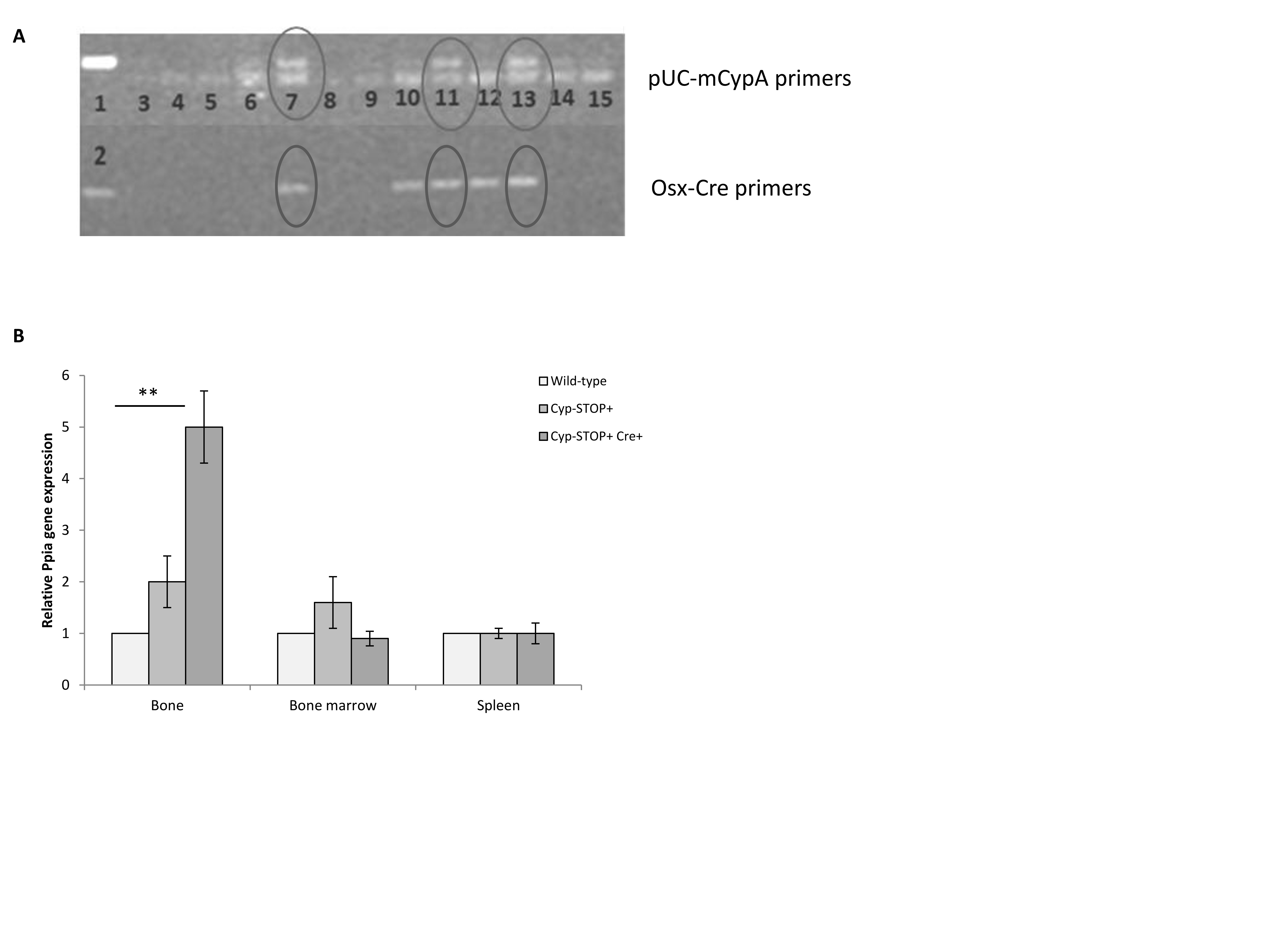

Supplement: Supplementary file 1 [file ijms-24-11279-s001.zip › supplementary-fig-S1.jpeg]

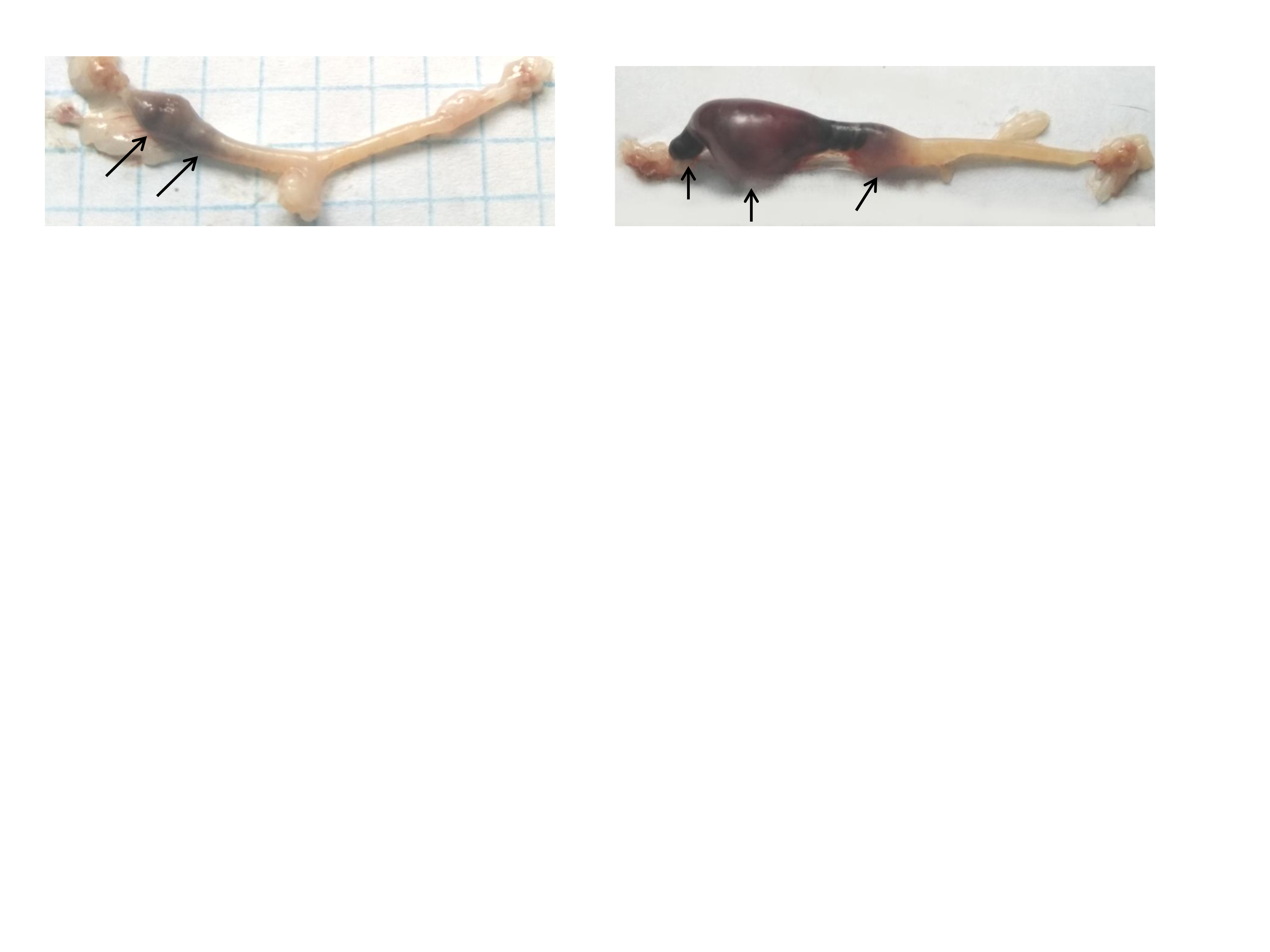

Supplement: Supplementary file 1 [file ijms-24-11279-s001.zip › supplementary-fig-S2.jpeg]

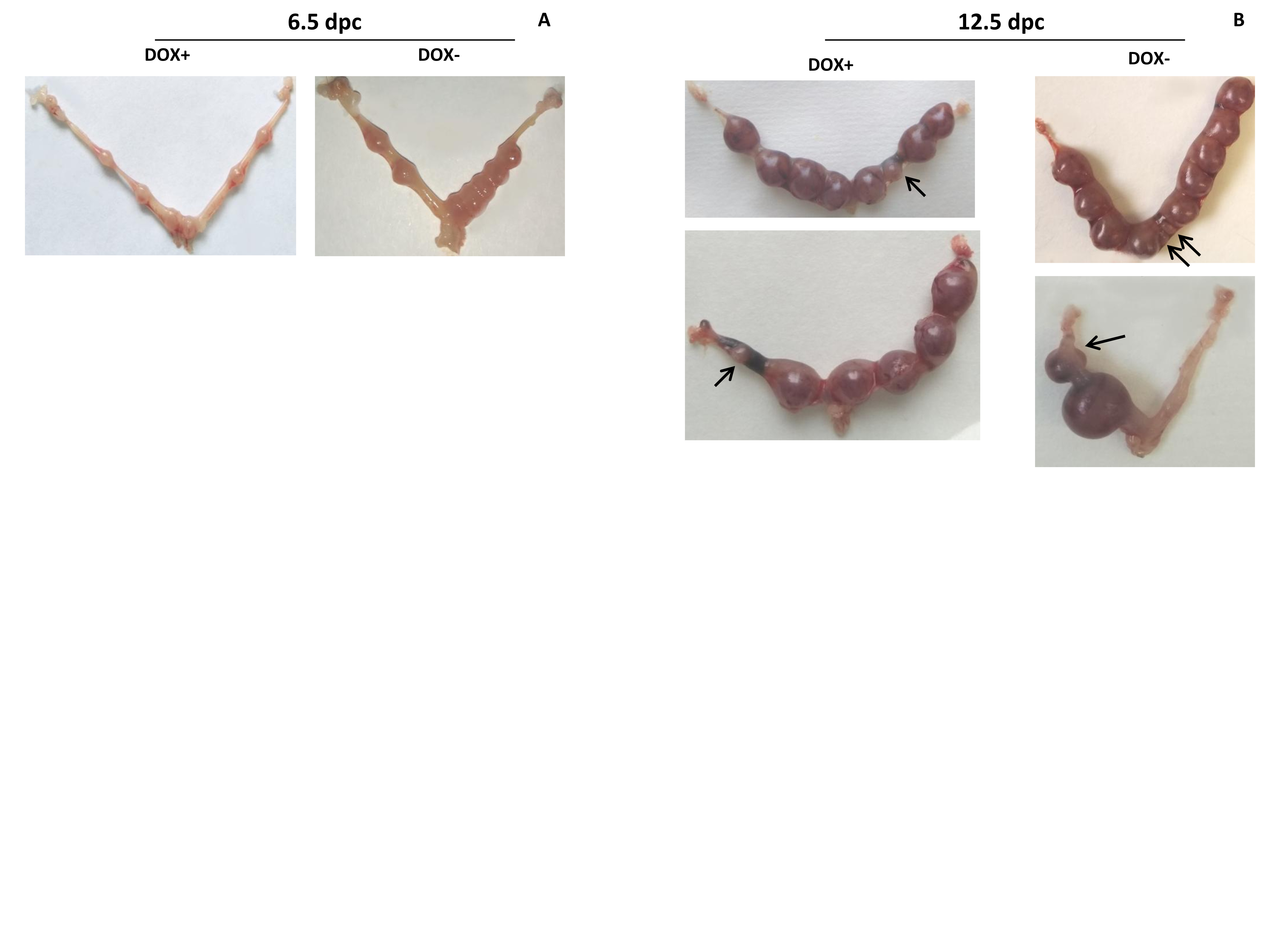

Supplement: Supplementary file 1 [file ijms-24-11279-s001.zip › supplementary-fig-S3.jpeg]

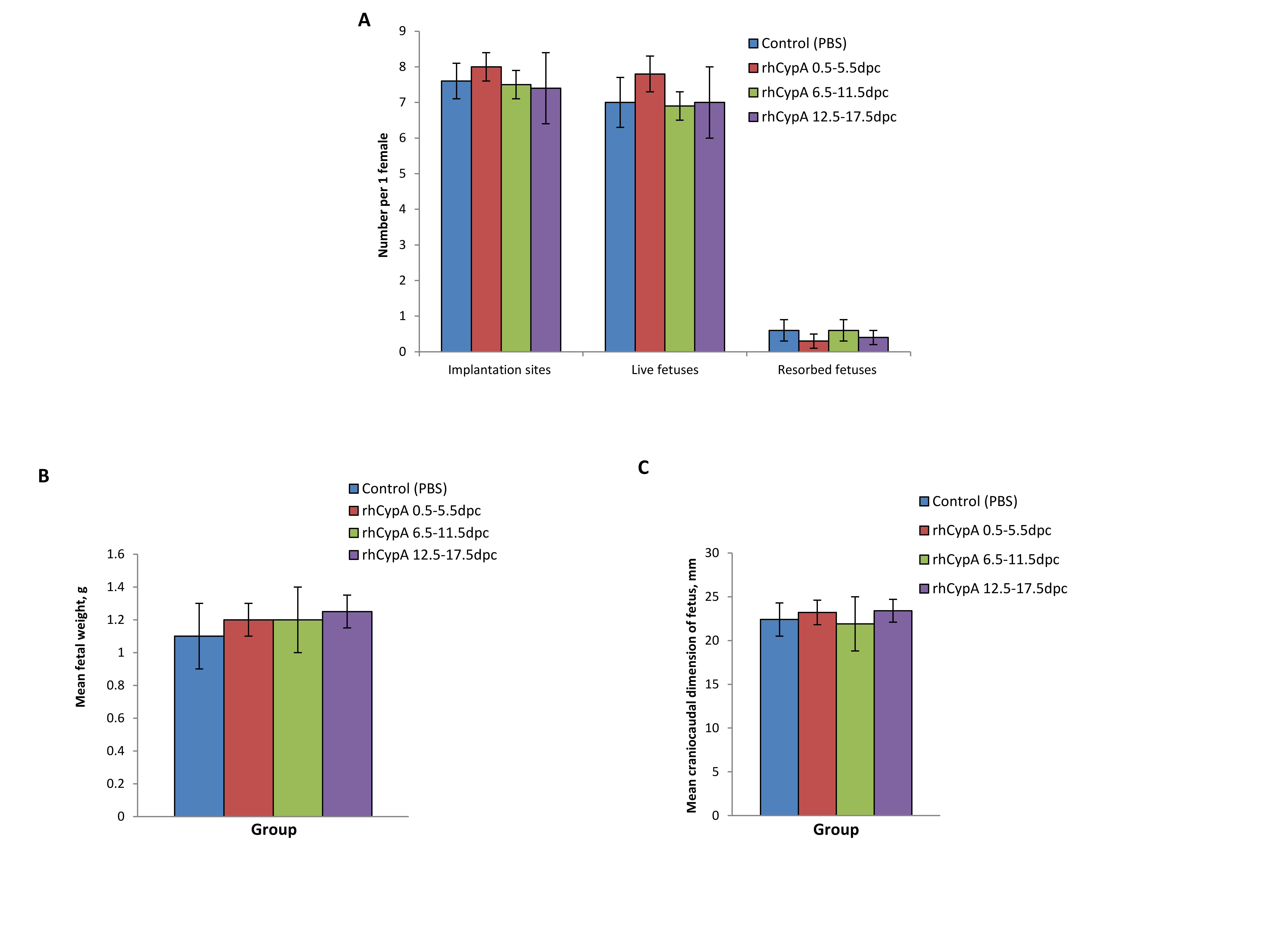

Supplement: Supplementary file 1 [file ijms-24-11279-s001.zip › supplementary-fig-S4.jpeg]

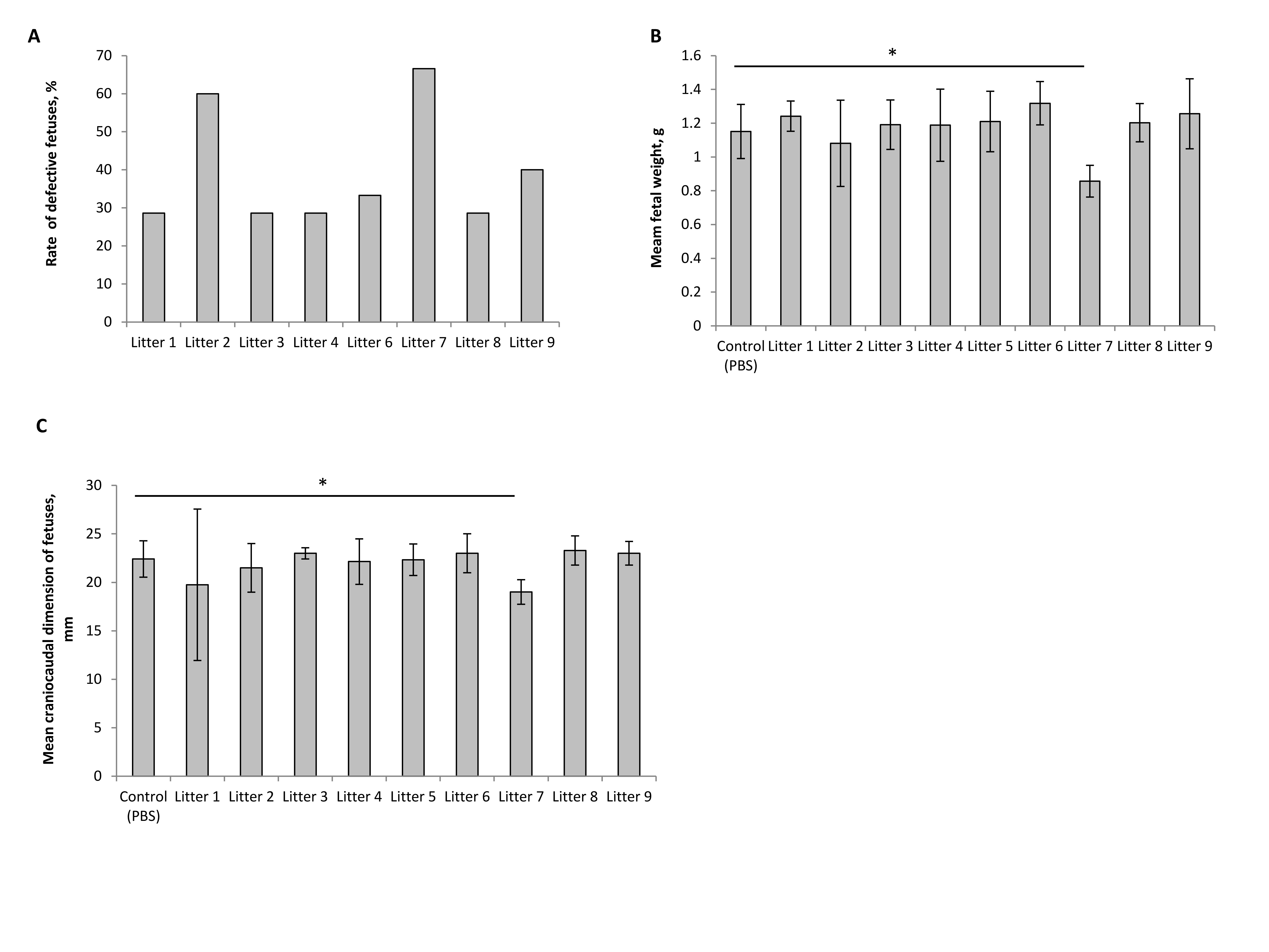

Supplement: Supplementary file 1 [file ijms-24-11279-s001.zip › supplementary-fig-S5.jpeg]

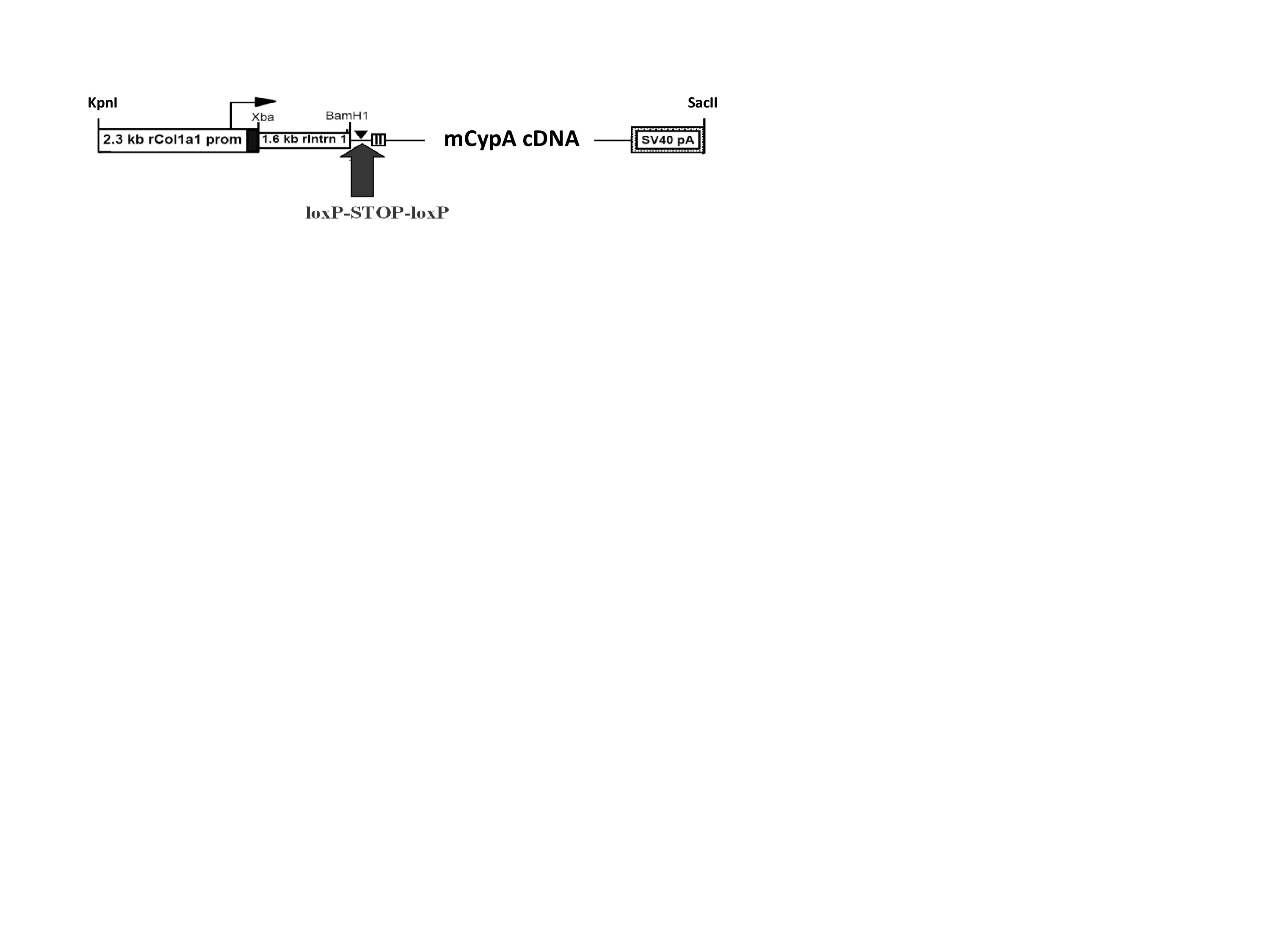

Supplement: Supplementary file 1 [file ijms-24-11279-s001.zip › supplementary-fig-S6.jpeg]

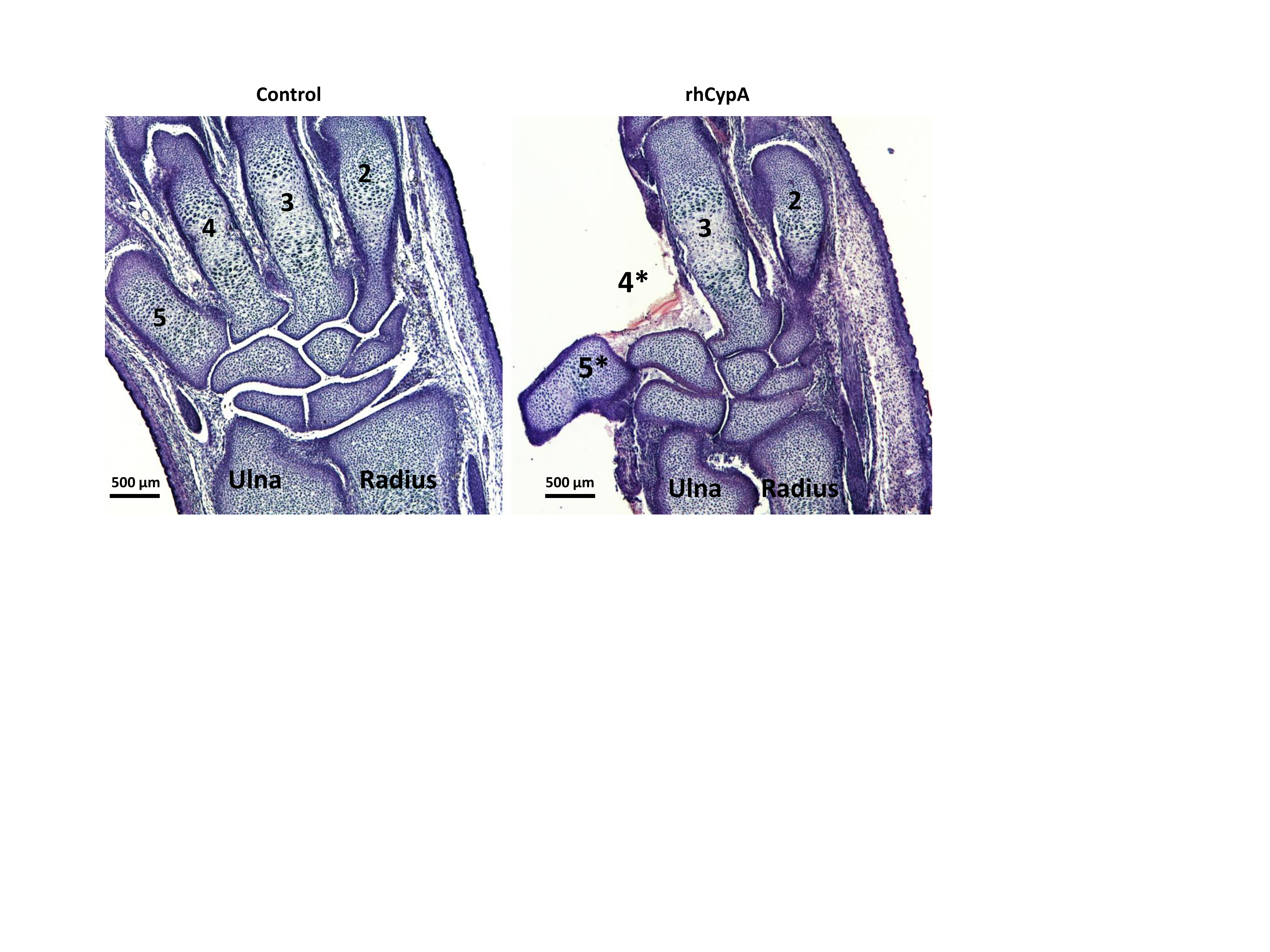

Supplement: Supplementary file 1 [file ijms-24-11279-s001.zip › supplementary-fig-S7.jpeg]
